# Identification of potential therapeutic targets in human head & neck squamous cell carcinoma

**DOI:** 10.1186/1758-3284-1-27

**Published:** 2009-07-14

**Authors:** Jing Han, Mitomu Kioi, Wei-Sing Chu, Jan L Kasperbauer, Scott E Strome, Raj K Puri

**Affiliations:** 1 Tumor Vaccines and Biotechnology Branch, Division of Cellular and Gene Therapies, Center for Biologics Evaluation and Research, Food and Drug Administration, Bethesda, MD 20892, USA; 2 Department of Scientific Laboratories, Armed Forces Institute of Pathology, Washington, DC 20306-6000, USA; 3 Mayo Clinic Cancer Center, 200 First Street Southwest, Rochester, Minnesota, 55905, USA; 4 Department of Otorhinolaryngology, Head & Neck Surgery, School of Medicine, University of Maryland, Baltimore, MD 21201, USA

## Abstract

**Background:**

Human head and neck squamous cell carcinoma (HNSCC) is an aggressive and recurrent malignancy. Identification of unique or overexpressed cell-associated or cell surface antigens is critical for diagnosis and development of cancer vaccines and targeted therapies for HNSCC. We have used high throughput microarray technology to search for candidate targets in HNSCC.

**Methods:**

Gene expression profiling in 17 HNSCC tumors and 3 normal tonsil tissues was performed by microarray. QRT-PCR analysis was performed to validate the microarray results. The five candidate genes were further characterized by immunohistochemical technique in surgical samples and tissue arrays.

**Results:**

A total of 192 up-regulated genes at statistical significance of *p *< 0.01 and log2 ratio ≥ 1 were identified in HNSCC tumors compared to normal tissues. These genes belong to immune response, cell growth, cell cycle regulation, oncogenes, metabolism and others. Five potential novel target genes (FABP5, CD24, CD44, CD74, and HSP27) were identified, which were highly expressed in HNSCC tumor samples and tissue arrays. CD24, CD44, and CD74 proteins were expressed on the cell surface, and FABP5 and HSP27 proteins were predominantly expressed in the cytoplasm of HNSCC.

**Conclusion:**

Five genes and their products may serve as a diagnostic biomarker or therapeutic target for HNSCC. While additional work is needed to elucidate the biological significance of these proteins, CD24 and CD74 expressed only in small proportion of cells indicating tumor heterogeneity and subtypes of tumor initiating cells (CD24+/CD44+) present in HNSCC.

## Background

In the United States, an estimated 40,000 new cases of head and neck cancers are diagnosed each year [1]. More than 90% of head and neck cancers are of squamous cell carcinoma, and arise from diverse anatomical locations, including lip/oral cavity, nasopharynx, oropharynx, larynx, and hypopharynx [1,2]. Although numerous studies have reported on the mechanism of oncogenesis, the precise cellular mechanism of tumor development, tumor progression, and tumor aggressiveness are not known. The survival rate of patients with head & neck squamous cell carcinoma (HNSCC) has not improved significantly despite multimodality therapy including surgery, radiation therapy, and chemotherapy. Recent investigations have focused on novel therapeutic approaches, and on the identification of molecular targets for therapy [3,4].

Microarray approaches have been widely used to identify genes associated with tumorigenesis, metastatic potential, clinically distinct subgroups of tumors, and prognostic biomarkers and potential targets for novel therapeutic agents. Identification of unique or overexpressed antigens or cell surface proteins is critical for the development of cancer vaccines and targeted immunotoxins or cytotoxins, which may offer alternative approach for caner therapy.

Previously, our group has identified Interleukin-13 receptor α2 (IL13Rα2) as a unique marker in human head and neck cancer, which can be used to distinguish a subset of HNSCC and may serve as a target for immunotoxin or cytotoxin therapies [5]. In attempt to identify novel therapeutic targets for treatments of HNSCC, in the present study, we analyzed differentially expressed genes in HNSCC tumors derived from clinical samples, and compared with normal tissues using microarray technology. The genes significantly up-regulated in HNSCC were selected as candidates for further evaluation. Based on the literature and availability of antibody, highly expressed genes (CD24, CD44, and CD74) encoding cell surface receptors and (FABP5 and heat shock protein Hsp27) encoding cytoplasmic proteins were evaluated at protein levels by IHC. The in-depth analysis of gene expression and protein expression in tissue arrays of these candidate genes revealed that they may serve as potential prognostic biomarkers or therapeutic targets for targeted therapy or antigen directed immunotherapy.

## Methods

### Tissue Sample Collection

A total of 17 HNSCC and 3 normal tissues (from tonsillectomies) from 20 patients were collected at Mayo Clinic Cancer Center. The Institutional Review Board (IRB) of the Mayo Clinic approved sample collection and RIHSC (Research Involving Human Subjects Committee) of the FDA had approved sample receipt at the FDA laboratory. Samples were immediately stabilized by freezing in liquid nitrogen in the operating room. All samples were pathologically confirmed. The clinical characteristics of the tissue samples are summarized in Table 1. At the FDA laboratory, the specimen was cut into two pieces, one piece for RNA isolation and another for paraffin embedding. H & E staining of Paraffin-embedded tissue sections were performed by Histoserv, Inc (Germantown, MD).

**Table 1:** Patient demographics and pathology of tumors

**Patient #**	**Sex**	**Age (yrs)**	**Tumor Location and Grade**	**History of ETOH or Smoking**	**Pathological Subtypes**
***Normal Tissues***					
A9	F	32	tonsillectomy	No	Follicular hyperplasia
B9	F	30	tonsillectomy	ETOH/Smoking	Follicular hyperplasia
C9	M	40	tonsillectomy	No	Follicular hyperplasia
					
***Tumors***					
A1	/	/	Base of tongue Grade 3	/	Primary SCCA
A6	/	/	Base of tongue Grade 3	/	Primary SCCA
E1	F	68	Base of tongue Grade 4	ETOH/Smoking	Recurrent SCCA
I1	M	38	Base of tongue	ETOH/Smoking	Primary SCCA
F1	F	65	Larynx, Grade 2	ETOH/Smoking	Primary SCCA
C1	M	53	Larynx, Grade 3	ETOH/Smoking	Primary SCCA
D1	F	40	Larynx, Grade 3	No	Metastatic, unknown primary
A3	/	/	Lymph node-neck	/	Primary SCCA
A5	/	/	Lymph node-neck	/	Primary SCCA
H1	F	44	Lymph node-neck, Grade 3	No	Primary SCCA
B1	M	72	Tonsil, Grade 3	No ETOH, Smoking	Primary SCCA
B2	M	60	Tonsil	ETOH/Smoking	Primary SCCA
B5	M	62	Tonsil	ETOH/Smoking	Primary SCCA
B4	M	53	Buccal mucosa	ETOH, Minimal Smoking	Recurrent SCCA
G1	M	72	Buccal mucosa	Minimal ETOH/Smoking	Primary SCCA
A2	M	60	Supraglottic	/	Primary SCCA
B3	F	72	Supraglottic Grade 4	ETOH/Smoking	Recurrent SCCA

/: patient information not available

SCCA: Squamous cell carcinoma

### Microarray Experiments

Total RNA from tissue samples used for microarray experiments were extracted by Trizol reagent according to the Manufacture's instructions (Invitrogen). Briefly, tissues were suspended in Trizol reagent (Invitrogen), homogenized, and RNA was isolated following the Manufacture's instructions. Human universal RNA (huRNA) (Strategene) was used as a common reference for all experiments. The human microarrays containing approximately 17,000 oligonucleotides were used which were produced in our laboratory. The detailed information regarding array printing, post-print processing, and testing array quality is described elsewhere [6]. Target preparation, microarray hybridization, image quantification, and data analyses have been described previously, with modifications [7]. In brief, 5 μg of total RNA was reverse-transcribed using 5'-amino-modified primers with amino-allyl-dUTP. cDNAs synthesized from HNSCC tissues samples were labeled with Cy5 dye, and cDNA from universal RNA labeled with Cy3 dye. Labeled and combined cDNA probes were denatured, mixed in SlideHyb #1 hybridization buffer (Ambion, Austin, TX), and placed onto microarray slides. Arrays were hybridized at 42°C in MAUI hybridization system (BioMicro Systems) for 16–18 hours, and then washed with 1 × SSC with 0.05% SDS for 4 min., 0.1 × SSC for 4 min. Slides were quickly spin-dried.

### Data Analysis and Statistics

Microarray slides were scanned on a GenePix 4000B scanner (Axon Instruments, Inc., Foster City, CA) with a 10 μm resolution. Scanned raw images were analyzed and data files were generated with GenePix Pro 5.1 (Axon) software. For analysis, data files were uploaded into mAdb (microarray database), and analyzed by the software tools provided by the Center for Information Technology (CIT), NIH. The advanced filters, spots size at least 30 μm, minimum fluorescent intensity of 100 in both Cy3 and Cy5 channels, were applied before data analysis. A standard global normalization approach was used for each experiment. All of the extracted data was normalized using a 50^th ^percentile (median) normalization method. Statistical analyses were performed and group comparison *t*-test was used to compare the difference of gene expression between normal and tumor samples. Genes were selected with the following criteria: *p *< 0.01, and log2 fold difference ≥ 1 in mean expression between HNSCC tumors and normal tissues.

### Gene-Specific Confirmation by QRT-PCR

QRT-PCR was performed on selected genes identified by gene expression profiling. The first-strand cDNA was synthesized from 1 μg of total RNA using Superscript II Reverse Transcriptase (Invitrogen, Carlsbad, CA) according to manufactures specifications. The resulting cDNA was amplified by using gene-specific primers. Primer sequences are as follows: *FABP5*, forward, 5'-agcagctggaaggaagat-3', and reverse, 5'-gatacaatctggcttggc-3'; *CD24*, forward, 5'-aggatgggattgtggaat-3', and reverse, 5'-attagtgccgtcgaaaca-3'; *EIF4G2*, forward, 5'-gcagaagatgcaccaaac-3', and reverse, 5'-atggctctctgttcctcc-3'; *KRT18*, forward, 5'-gtagatgcccccaaatct-3', and reverse, 5'-cactgtggtgctctcctc-3'; *LGALS1*, forward, 5'-tggactcaatcatggctt-3', and reverse, 5'-ggttgttgctgtctttgc-3'; *RPLP0*, forward, 5'-gacggattacaccttccc-3', and reverse, 5'-tggcttcaaccttagctg-3'; *GAPDH*, forward 5'-aaggtgaaggtcggagtcaa-3', and reverse 5'-gatctcgctcctggaagatg-3'. The specificity of primers was first verified by RT-PCR and gel electrophoresis. QRT-PCR reactions were performed with SYBR Q-PCR master mixture (Stratagene, La Jolla, CA) following the manufactures protocol, and reactions were carried out in a Stratagene Mx3000P machine. Buffer and no template controls were included in each assay run. All samples and controls were run in triplicate.

### Immunohistochemistry of HNSCC

Immunohistochemical studies of selected genes were performed on both formalin-fixed, paraffin-embedded tissue sections from patients and HNSCC tissues arrays, which were purchased from Biomax Inc. Tissue diagnosis of viable tissue was confirmed by H and E staining for squamous cell carcinoma of head and neck. HNSCC tissue arrays contained 16 cases of primary HNSCC and 8 cases of cancer adjacent normal tissues in duplicate and mounted on the slides. Tissue sections or tissue arrays were deparaffinized by xylene, and then, re-hydrated with sequential washes of 100%, 75%, and 50% ethanol, and PBS. For antigen retrieval, slides were placed in 50 mM citrate buffer pH6.0 (Vector Lab, CA), heated in a microwave oven for 5 min, and then stayed in the buffer for 15 min. Endogenous peroxidase activity was inhibited with 3% hydrogen peroxidase in PBS. Non-specific binding was blocked with 2.5% normal serum and 1% bovine serum albumin (BSA) for 1 hr. Tissue sections and tissue arrays were then incubated with various antibodies, CD24, CD44, and HSP27 (Chemicon; Temecular, CA), CD74 (Santa Cruz Biotech; Santa Cruz, CA), FABP5 (ProteinTech; Chicago, IL), or isotype control (IgG) (Sigma) overnight at 4°C. Immunodetection was performed using ABC staining systems according to manufacturer's instructions (Santa Cruz Biotech; Santa Cruz, CA). All sections were counterstained with haematoxylin. After dehydration with washes of 95% and 100% ethanol and xylene, tissue sections and tissue arrays with permanent mounting medium were covered with glass coverslips, and viewed by light microscope.

## Results

### Identification of Differentially Expressed Genes in HNSCC

The study population was representative of the general population with HNSCC, having a median age of 60 years at presentation. All 17 tumor tissues were squamous cell carcinoma from different locations, and all three tonsil samples showed follicular hyperplasia (Table 1). Gene expression profiling was performed in all tumor and normal tissues. In order to identify the potential therapeutic targets in HNSCC, we mainly focused on the up-regulated genes in HNSCC. A total of 192 genes, which were significantly up-regulated (with *p *< 0.01 and log2 fold at least ≥ 1) in HNSCC tumors compared with normal tonsil tissues, were identified. A number of up-regulated genes were classified, which belong to various biological processes including cell growth and proliferation, protein translation and synthesis, metabolism, signaling, and immune response (Table 2, and see Additional file 1).

**Table 2:** Selected up-regulated genes identified in HNSCC*

**GeneBank Access ID**	**Gene Symbol & Annotation**	Log2 Fold Difference**
***Immune Response***		
L19686	MIF, macrophage migration inhibitory factor	3.1
M13560	CD74, CD74 antigen	2.6
M63438	IGKC, immunoglobulin kappa constant	1.9
J03909	IFI30, interferon, gamma-inducible protein 30	1.5
AJ251549	IL26, interleukin 26	1.4
L33930	CD24, CD24 antigen	1.4
X16302	IGFBP2, insulin-like growth factor binding protein 2	1.4
S75725	IGFBP7, insulin-like growth factor binding protein 7	1.4
		
***Cell Growth, Maintenance/Cell cycle Regulation***		
Y00503	KRT19, keratin 19	3.9
U43901	LAMR1, laminin receptor 1	3.2
AL031670	FTL, ferritin, light polypeptide	2.5
J00124	KRT14, keratin 14	2.5
X07696	KRT 15, keratin 15	2.4
X95404	CFL1, cofilin 1	2.3
D13627	CCT8, chaperonin subunit 8	2.1
AF026291	CCT4, chaperonin subunit 4	2.0
Z68228	JUP, junction plakoglobin	1.9
M26326	KRT18, keratin 18	1.8
L42583	KRT6C, keratin 6C	1.7
		
***Translation and Protein Synthesis***		
AK001313	RPLP0, ribosomal protein LP0	4.1
L06499	RPL37A, ribosomal protein L37A	3.9
U73824	EIF4G2, translation initiation factor 4 gamma 2	3.2
M64241	RPL10, ribosomal protein L10	3.2
X69150	RPS18, ribosomal protein S18	3.1
M84711	RPS 3A, ribosomal protein S3A	3.0
NM_000996	RPL35A, ribosomal protein L35A	3.0
U25789	RPL21, ribosomal protein L21	2.9
L11566	RPL18, ribosomal protein L18	2.8
Z21507	EEF1D, eukaryotic translation elongation factor 1D	2.3
AL117412	EIF4A2, eukaryotic translation initiation factor 4A	2.2
AC002544	EIF3S8, eukaryotic translation initiation factor 3, subunit 8	2.1
		
***Metabolism***		
Z23090	HSPB1, heat shock 27 kDa protein	4.2
M94856	FABP5, fat acid binding protein 5	3.7
NM_021130	PPIA, peptidylprolyl isomerase A (cyclophilin A)	2.9
M26252	PKM2, pyruvate kinase, muscle	2.6
NM_001679	ATP1B3, ATP synthase Na+/K+ transporting, beta 3	2.6
AF061735	ATP5H, ATP synthase H+ transporting subunit	2.1
Y00483	GPX1, glutathione peroxidase 1	2.0
AL021546	COX6A1, cytochrome c oxidase subunit VI a1	1.9
X13923	COX6B, cytochrome c oxidase subunit VI b	1.8
X13794	LDHB, lactate dehydrogenase B	1.7
Z85996	CDKN1A, cyclin-dependent kinase inhibitor 1A	1.7
M60483	PPP2CA, protein phosphatase 2 catalytic subunit	1.7
Y13936	PPM1G, protein phosphatase 1G	1.6
U09813	ATP5G3, ATP synthase H+ transporting subunit	1.6
D29011	PSMB5, proteasome subunit, beta 5	1.6
AF047181	NDUFB5, NADH dehydrogenase beta subcomplex 5	1.5
		
***Ion Binding Proteins***		
Y07755	S100A2, S100 calcium binding protein A2	3.6
D38583	S100A11, S100 calcium binding protein A11	2.4
NM_020672	S100A14, S100 calcium binding protein A14	1.9
X99920	S100A13, S100 calcium binding protein A13	1.2
		
***Others***		
M14328	ENO1, enolase 1	3.3
M26880	UBC, ubiquitin C	3.2
S54005	TMSB10, thymosin, beta 10	2.8
D87953	NDRG1, M-myc downstream regulated gene 1	2.4
M36981	NME2, non-metastatic cells 2 protein	2.3
AF055008	GRN, granulin	2.3
X57348	SFN, stratifin	2.3
U46751	SQSTM1, sequestosome 1	2.2
X67951	PRDX1, peroxiredoxin 1	2.1
X65607	MT1X, metallothionein 1X	1.5
X84709	FADD, Fas associated death domain	1.2

* All selected genes with *p*-value < 0.01.

**Log2 ratio fold-differences between tumors and normal tissues were determined by subtracting the ratio of genes in normal tissue versus uRNA from the ratio of genes in HNSCC tissues versus uRNA. (Log2 ratio of 1 equals to 2-fold difference between HNSCC and normal tissue; Log2 ratio of 2 equals to 4-fold difference between HNSCC and normal tissue, and so on)

A prominent gene expression signature up-regulated in HNSCC includes genes associated with inflammation and immune response, such as MIF, CD74 and CD24, which were not previously identified in HNSCC (Table 2). Another prominent signature of up-regulated genes includes FABP5, HSP27, S100A2, EIF4G2, and RPLP0, which are associated with various biological functions such as metabolism, ion binding, and protein translation and synthesis (Table 2).

**Figure 1:**
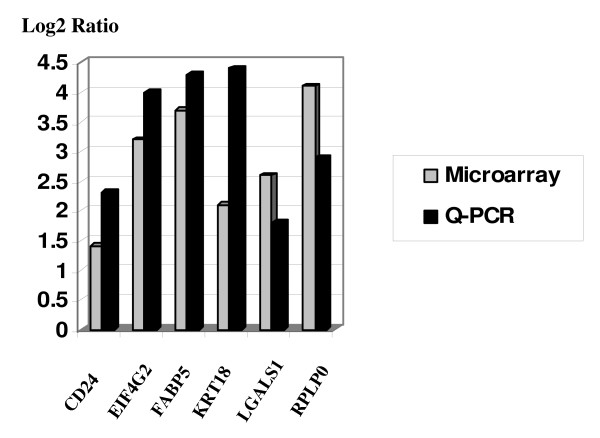
**Real-Time PCR confirmation of selected genes**. The open bar represents expression ratio compared to huRNA control from microarray experiments, and solid bar presents the ratio from QRT-PCR experiments. Results indicate similar trend of gene expression in both microarray and QRT-PCR experiments.

To confirm over expressed genes identified by microarray in HNSCC, six genes including CD24, FABP5, EIF4G2, LGALS1, KRT18, and RPLP0, were selected and QRT-PCR analysis was performed. The log-transformed measurement of gene expression levels determined by microarray positively correlated with the QRT-PCR analysis (Fig 1). Both microarray and QRT-PCR results confirmed similar trend of gene expression profile of selected genes in the HNSCC.

### Expression of Selected Gene Products in HNSCC

**Figure 2:**
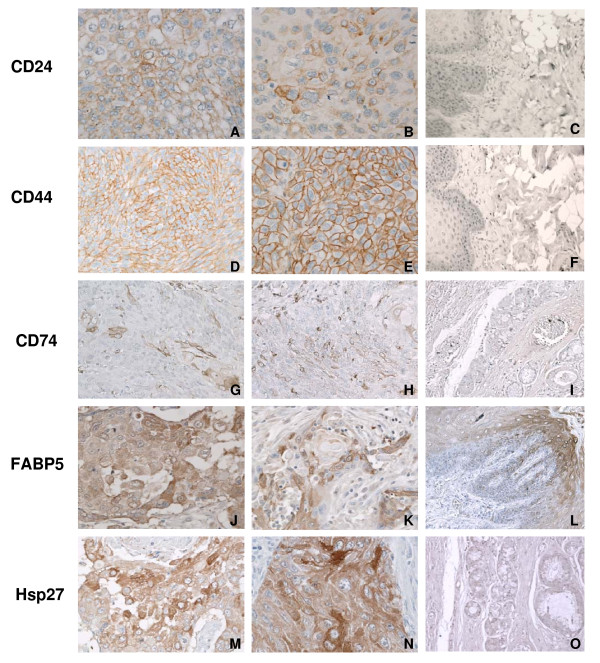
**Immunohistochemical analysis of CD24, CD44, CD74, FABP5, and Hsp27 in HNSCC samples**. HNSCC tissue arrays contained 16 tumor samples from various locations and different stages. A and B are tumor samples and stained for CD24. D and E stained for CD44, and showed strong positive reactions in most tumor cells. Both CD24+ and CD44+ show cell surface staining, and CD24+ cells present in a small cluster of cells in the large tumor mass. (Magnification: ×200). G and H stained for CD74. Some of tumor cells showed CD74+. J and K stained for FABP5, and M and N stained for Hsp27. FABP5 shows strong cytoplasmic staining as well as Hsp27. C, F, I, L, O: tumor adjacent normal tissue. CD24, CD44, CD74, FABP5, and Hsp27 were negative in normal tissues, although FABP5 shows positive staining in the basal layer of dermis (Fig 2L), but negative in other areas; (Magnification: ×200). IgG was used as a negative control (Data not shown).

To narrow down the range of potential therapeutic targets in 192 up-regulated genes identified in our HNSCC samples, we mainly focused on the genes encoding cell surface receptors and some cytoplasmic proteins. Among these genes, we selected five gene products (CD24, CD44, CD74, FABP5, and HSP27) as antibodies to these products were available for paraffin embedded tissue sections. The expression of selected gene products was confirmed by IHC in tissue sections and tissue arrays. Since CD24, CD44, and CD74 are cell surface proteins and function as adhesion molecules, their expression was predominantly on the cell surface. Nine out of 16 HNSCC tumors in tissue arrays were positive for CD24 expression. However, its expression was found only in small clusters of cells within tumors (Fig 2A and 2B). CD44 was strongly expressed on the cell surface of all tumor cells in all cases of HNSCC samples (Fig 2D and 2E). CD74 positive cells were also identified in HNSCC tissue arrays (Fig 2G and 2H). In contrast, CD24, CD44, or CD74 positive cells were not detected in adjacent normal tissues present in tissue arrays (Fig 2C, F, and 2I).

As FABP5 and HSP27 are intra cytoplasmic proteins, their expression was examined in the cytoplasmic compartment. Both proteins showed strong positive cytoplasmic staining in majority of cases in the HNSCC tissue arrays (Fig 2J and 2K, M and 2N). In contrast, only background staining was observed for either FABP5 or HSP27 in the adjacent normal tissues (Fig 2L and 2O), except the basal layer of dermis in normal tissue also showed FABP5 positive staining (Fig 2L). Isotype control antibody showed no staining in any section (Data not shown).

## Discussion

Global gene expression profiling studies of human HNSCC tumors have identified genes associated with tumorigenesis and metastatic potential [8-13]. In addition, molecular classification of HNSCC has also been proposed based on gene expression patterns [11-13]. It has been reported that gene expression profile can distinguish clinically distinct subgroups of HNSCC, identify genes potentially associated with advanced-stage of the disease, and predict outcome of treatment [9,11,14]. In our study, in order to identify novel therapeutic targets for diagnosis or treatments, we compared gene expression in HNSCC with normal tissues and further characterized protein expression of over expressed genes in HNSCC samples. We identified 192 up-regulated genes in HNSCC compared with normal tissues. Based on our results, published literature, and availability of antibody, five candidate genes (CD24, CD44, CD74, FABP5, and HSP27) were selected for further characterization in HNSCC samples. These gene products were expressed in most tumor samples but not in normal tissues. Although various studies have reported on the expression of these genes in various tissues, our study presents their significance in HNSCC tumors.

Among selected genes, CD24, CD44, and CD74 are cell surface adhesion molecules shown to be over expressed in various cancers [15-21]. Among these, CD44 was most strongly expressed in all HNSCC samples in tissue arrays. Interestingly, CD44+ subpopulation of cells with cancer stem cell properties has been identified in HNSCC and various other cancer types [21-25]. CD24 has also been shown to be expressed in various tumors. Its expression has been used as a prognostic indicator of poor survival in breast cancer, non-small cell lung carcinoma, and prostate cancers [15-18]. However, in contrast to CD44, only a small portion of CD24+ cells were positive in HNSCC cancer cells in our study. These results suggest that a subset of CD24+/CD44+ cells exists, which may represent cancer stem cells in HNSCC. This hypothesis is supported by a report which showed that a highly tumorigenic subpopulation of pancreatic cancer cells express cell surface markers CD44, CD24, and epithelial-specific antigen (ESA) [23]. This subpopulation of pancreatic cancer cells with CD44+/CD24+/ESA+ phenotype (only 0.2 to 0.8% of pancreatic cancer cells) had a 100-fold increased tumorigenic potential compared with other cancer cells [23]. Future studies will focus on the isolation and characterization of the CD24+/CD44+ and other marker positive tumor subpopulations from HNSCC.

CD74 is another cell surface membrane protein identified in this study, which was not previously reported in HNSCC. However, the over expression of CD74 has been reported in many different malignancies, including gastric tumors, renal epithelial neoplasms, pancreatic cancers, certain types of sarcoma and skin cancer, and non-small cell lung cancers [26-30]. CD74 has been identified as the high-affinity receptor for the macrophage migration inhibitory factor [31]. The over expression of macrophage migration inhibitory factor has also been reported in various cancers, including lung cancer, breast cancers, prostate cancers, ovarian cancers, colorectal cancers, and HNSCC tumors in this study (Table 2) [30,32-35]. Overexpression of tumor cell-derived macrophage migration inhibitory factor (MIF) in solid tumors is related to tumor growth, progression, and angiogenesis [32-34,36]. Recent study on non-small cell lung cancers suggested that the co-expression of MIF and its receptor CD74 is associated with greater tumor vascularity and greater of angiogenic CXC chemokines [30]. In vitro inhibition of MIF or its receptor resulted in reduced production of angiogenic CXC chemokines by lung cancer cells [30]. In addition, the clinical study in colorectal cancer patients found that the serum level of MIF was significantly increased in cancer patients, suggesting that MIF could be used as a diagnostic marker in colorectal cancers [35]. In our study, since both MIF and its receptor CD74 are highly expressed in HNSCC, these results suggest that both MIF and CD74 may potentially serve as valuable biomarkers in HNSCC. In addition, as a cell membrane protein, CD74 may serve as a new target for anti-cancer therapy of HNSCC. MIF, on the other hand, may serve as a new diagnostic marker for HNSCC.

FABP5 is a fatty acid-binding protein and is expressed in epidermis and endothelial cells of the microvasculature of different organs [37]. FABP5 has also been identified as a tumor-associated antigen, which is highly expressed in various cancers [38,39]. FABP5 was detected in the sera of HNSCC patients with early stage cancer [40]. Antibodies specific for FABP5 were significantly increased in a substantial amount in patients, suggesting that FABP5 may be a potential diagnostic biomarker for HNSCC [40]. As FABP5 is highly expressed at both mRNA and protein levels in HNSCC compared to normal tissues, our results support previous suggestion that FABP5 may serve as a biomarker for HNSCC.

Another interesting gene identified in HNSCC is heat shock protein (HSP27). Over expression of HSP27 in HNSCC was not reported previously, although HSPs have been reported to be over-expressed in a wide range of human cancers, which suggest that HSPs may serve as an effective target for therapy [41,42]. In addition, over expression of HSP has been correlated with a poor prognosis in terms of survival and response to therapy in specific cancer types [41,43,44]. HSP27 is associated with poor prognosis in gastric cancer, liver cancer, prostate carcinoma, and non-small cell lung carcinoma [41,44]. HSP27 is also implicated in resistance to chemotherapy in breast cancer [41]. Several small-molecule drugs that target the HSP27 have been identified as potential anticancer agents [45]. Thus, up-regulation of HSP27 in HNSCC tumors identified in our current study provides opportunities for a new biomarker of disease monitoring and development of new targeted therapy for HNSCC.

## Conclusion

In this study, global gene expression profiling approach has been used to identify a large number of differentially expressed genes in HNSCC tumors compared to normal tissues. Among differentially expressed genes, we characterized five potential valuable targets for therapy and monitoring. These genes are expressed in the primary tumors. Future studies will be focused on the expression of these target genes in primary and metastatic lesions of HNSCC, and the impact of conventional therapies, such as chemotherapy or radiotherapy on the expression of these genes. While additional work is ongoing to elucidate the biological significance, our results suggest that CD24, CD44, CD74, and HSP27 may serve as new valuable therapeutic targets for the treatment of HNSCC; and FABP5 and MIF may be potential diagnostic markers for HNSCC. Our results also demonstrate that there may be a potentially new subpopulation (CD24+/CD44+) of tumor initiating cells in HNSCC.

## Competing interests

The authors declare that they have no competing interests.

## Authors' contributions

JH carried out the experimental work, data analysis and drafted the manuscript. MK and WSC carried out some experiments. JLK and SES provided the clinical samples and participated in the study design. RKP conceived and designed the study, performed data analysis and revised drafts and presentation of the manuscript and negotiated publication of this article. All authors read and approved the final manuscript.

## Supplementary Material

Additional file 1 **Gene expression changes in HNSCC compared to normal tonsils**. Data show up-regulated genes, with *p*-value < 0.01 and log2 ratio ≥ 1, in HNSCC compared with normal tonsil tissues. Click here for file

## References

[B1] Marur S, Forastiere AA (2008). Head and neck cancer: changing epidemiology, diagnosis, and treatment. Mayo Clin Proc.

[B2] Argiris A, Karamouzis MV, Raben D, Ferris RL (2008). Head and neck cancer. Lancet.

[B3] Leibowitz MS, Nayak JV, Ferris RL (2008). Head & neck cancer immunotherapy: clinical evaluation. Curr Oncol Rep.

[B4] Shurau K, O'Brien PE (2008). Molecular targets in squamous cell carcinoma of the head and neck. Curr Treat Opt Oncol.

[B5] Kawakami M, Kawakami K, Kasperbauer JL, Hinkley LL, Tsukuda M, Strome SE, Puri RK (2003). Interlukin-13 receptor a2 chain in human head and neck cancer serves as a unique diagnostic marker. Clin Cancer Res.

[B6] Yang AX, Mejido J, Bhattacharya B, Petersen D, Han J, Kawasaki ES, Puri RK (2006). Analysis of the quality of contact pin fabricated oligonucleotide microarrays. Mol BioTechnol.

[B7] Han J, Lee H, Nguyen NY, Beaucage SL, Puri RK (2005). Novel multiple 5'-amino modified primer for DNA microarrays. Genomics.

[B8] Järvinen AK, Autio R, Haapa-Paananen S, Wolf M, Saarela M, Grénmam R, Leivo I, Kallioniemi O, Mäkitie AA, Monni O (2006). Identification of target genes in laryngeal squamous cell carcinoma by high-resolution copy number and gene expression microarray analyses. Oncogene.

[B9] Cromer A, Carles A, Millon R, Ganguli G, Chalmel F, Lemaire F, Young J, Dembele D, Thibault C, Muller A, Poch O, Abecassis J, Wasylyk B (2004). Identification of genes associated with tumorigenesis and metastatic potential of hypopharyngeal cancer by microarray analysis. Oncogene.

[B10] Jeon GA, Lee JS, Patel V, Gutkind JS, Thorgeirsson SS, Kim EC, Chu IS, Amornphimoltham P, Park MH (2004). Global gene expression profiles of human head and neck squamous carcinoma cell lines. Int J Cancer.

[B11] Ginos MA, Page GP, Michalowicz BS, Patel KJ, Volker SE, Pambuccian SE, Ondrey FG, Adama GL, Gaffney PM (2004). Identification of a gene expression signature associated with recurrent disease in squamous cell carcinoma of the head and neck. Cancer Res.

[B12] Belbin TJ, Singh B, Barber I, Socci N, Wenig B, Smith R, Prystowsky MB, Childs G (2002). Molecular classification of head and neck squamous cell carcinoma using cDNA microarrays. Cancer Res.

[B13] Chung CH, Parker JS, Karaca G, Wu J, Funkhouser WK, Moore D, Butterfoss D, Xiang D, Zanation A, Yin X, Shockley WW, Weissler MC, Dressler LG, Shores CG, Yarbrough WG, Perou CM (2004). Molecular classification of head and neck squamous cell carcinomas using patterns of gene expression. Cancer Cell.

[B14] Pramana J, Brekel MWM Van den, Velthysen MLF, Wessels LFA, Nuyten DS, Hofland I, Atsma D, Pimentel N, Hoebers FJP, Rasch CRN, Begg AC (2007). Gene expression profiling to predict outcome after chemoradiation in head and neck cancer. Int J Radiat Oncol Biol Phys.

[B15] Kristiansen G, Schluns K, Yongwei Y, Denkert C, Dietel M, Peterson I (2003). CD24 expression is a new prognostic marker in breast cancer. Clin Cancer Res.

[B16] Kristiansen G, Schluns K, Yongwei Y, Denkert C, Schluns K, Dietel M, Peterson I (2003). CD24 is an independent prognostic marker of survival in nonsmall cell lung cancer patients. Br J Cancer.

[B17] Kristiansen G, Pilarsky C, Pervan J, Sturzbecher B, Stephan C, Jung K, Loening S, Rosenthal A, Dietel M (2004). CD24 expression is a significant predictor of PSA relapse and poor prognosis in low grade or organ confined prostate cancer. Prostate.

[B18] Baumann P, Cremers N, Kroese F, Orend G, Chiquet-Ehrismann R, Uede T, Yagita H, Sleeman J (2005). CD24 expression causes the acquisition of multiple cellular properties associated with tumor growth and metastasis. Cancer Res.

[B19] Stein R, Mattes MJ, Cardillo TM, Hansen HJ, Chang CH, Burton J, Govindan S, Goldenberg DM (2007). CD74: A new candidate target for the immunotherapy of B-cell neoplasms. Clin Cancer Res.

[B20] Lazova R, Moynes R, May D, Scott G (1997). A marker to distinguish atypical fibroxanthoma from malignant fibrous histocytoma. Cancer.

[B21] Prince ME, Sivanandan R, Kaczorowski A, Wolf GT, Kaplan MJ, Dalerba P, Weissman IL, Clarke MF, Ailles LE (2007). Identification of a subpopulation of cells with cancer stem cell properties in head and neck squamous cell carcinoma. PNAS.

[B22] Bapat SA, Mali AM, Koppikar CB, Kurrey NK (2005). Stem and progenitor-like cells contribute to the aggressive behavior of human epithelial ovarian cancer. Cancer Res.

[B23] Li C, Heidt DG, Dalerba P, Burant CF, Zhang L, Adsay V, Wicha M, Clarke MF, Simeone DM (2007). Identification of pancreatic cancer stem cells. Cancer Res.

[B24] Collins AT, Berry PA, Hyde C, Stower MJ, Maitland NJ (2005). Prospective identification of tumorigenic prostate cancer stem cells. Cancer Res.

[B25] Ponti D, Costa A, Zaffaroni N, Pratesi G, Petrangolini G, Coradini D, Pilotti S, Pierotti MA, Daidone MG (2005). Isolation and in vitro propagation of tumorigenic breast cancer cells with stem/progenitor cell properties. Cancer Res.

[B26] Ishigami S, Natsugoe S, Tokuda K, Nakajo A, Iwashige H, Aridome K, Hokita S, Aikou T (2001). Invariant chain expression in gastric cancer. Cancer Lett.

[B27] Young AN, Amin MB, Moreno CS, Lim SD, Cohen C, Petros JA, Marshall FF, Neish AS (2001). Expression profiling of renal epithelial neoplasms: a method for tumor classification and discovery of diagnostic molecular markers. Am J Pathol.

[B28] Hustinx SR, Cao D, Maitra A, Sato N, Martin ST, Sudhir D, Iacobuzio-Donahue C, Cameron JL, Yeo CJ, Kern SE, Goggins M, Mollenhauer J, Pandey A, Hruban RH (2004). Differentially expressed genes in pancreatic ductal adenocarcinomas identified through serial analysis of gene expression. Cancer Biol Ther.

[B29] Cooper JZ, Newman SR, Scott GA, Brown MD (2005). Metastasizing atypical fibroxanthoma (cutaneous malignant histocytoma): report of five cases. Dermatol Surg.

[B30] McClelland M, Zhao L, Carskadon S, Arenberg D (2009). Expression of CD74, the receptor for macrophage migration inhibitory factor, in non-small cell lung cancer. Am J Path.

[B31] Leng L, Metz CN, Fang Y, Xu J, Donnelly S, Baugh J, Delohery T, Chen Y, Mitchell RA, Bucala R (2003). MIF signal transduction initiated by binding to CD74. J Exp Med.

[B32] Xu X, Wang B, Ye C, Yao C, Lin Y, Huang X, Zhang Y, Wang S (2008). Overexpression of macrophage migration inhibitory factor induces angiogenesis in human breast cancer. Cancer Lett.

[B33] Meyer-Siegler KL, Iczkowski KA, Leng L, Bucala R, Vera PL (2006). Inhibition of macrophage migration inhibitory factor or its receptor (CD74) attenuates growth and invasion of DU-145 prostate cancer cells. J Immunol.

[B34] Hagemann T, Robinson SC, Thompson RG, Charles K, Kulbe H, Balkwill FR (2007). Ovarian cancer cell-derived migration inhibitory factor enhances tumor growth, progression, and angiogenesis. Mol Cancer Ther.

[B35] Lee H, Rhee H, Kang HJ, Kim HS, Min BS, Kim NK, Kim H (2008). Macrophage migration inhibitory factor may be used as an early diagnostic marker in colorectal carcinomas. Am J Clin Path.

[B36] Nishihirs J, Ishibashi T, Fukushima T, Sun B, Sato Y, Todo S (2003). Macrophage migration inhibitory factor (MIF): its potential role in tumor growth and tumor-associated angiogenesis. Ann N Y Acad Sci.

[B37] Masouye J, Hagens G, Van Kuppevelt TH, Madsen P, Saurat JH, Veerkamp JH, Pepper MS, Siegenthaler G (1997). Endothelial cells of the human microvasculature express epidermal fatty acid-binding protein. Circ Res.

[B38] Zimmerman AW, Veerkamp JH (2002). New insights into the structure and function of fatty acid-binding proteins. Cell Mol Life Sci.

[B39] Adamson J, Morgan EA, Beesley C, Mei Y, Foster CS, Fujii H, Rudland PS, Smith PH, Ke Y (2003). High-level expression of cutaneous fatty acid-binding protein in prostatic carcinomas and its effect on tumorigenicity. Oncogene.

[B40] Rauch J, Ahlemann M, Schaffrik M, Mack B, Ertongur S, Andratschke M, Zeidler R, Lang S, Gires O (2004). Allogenic antibody-mediated identification of head and neck cancer antigens. Biochem Biophy Res Comm.

[B41] Ciocca DR, Calderwood SK (2005). Heat shock proteins in cancer: diagnostic, prognostic, predictive, and treatment implications. Cell Stress Chaperones.

[B42] Soti C, Nagy E, Giric Z, Vigh L, Csermely P, Ferdinandy P (2005). Heat shock proteins as emerging therapeutic targets. Br J Pharmacol.

[B43] Pick E, KLuger Y, Giltnane JM, Moeder C, Camp RL, Rimm DL, Kluger HM (2007). High HSP90 expression is associated with decreased survival in breast cancer. Cancer Res.

[B44] Malusecka E, Krzyzowska GS, Gawrychowski J, Fiszer KA, Kolosza Z, Krawczyk Z (2008). Stress proteins HSP27 and HSP70 predict survival in non-small cell lung carcinoma. Anticancer Res.

[B45] Hadaschik BA, Jackson J, Fazli L, Zoubeidi A, Burt HM, Gleave ME, So AI (2008). Intravesically administered antisense oligonucleotides targeting heat-shock protein-27 inhibit the growth of non-muscle-invasive bladder cancer. BJU Int.

