# Supplementary material for: Identification of potential therapeutic targets in human head & neck squamous cell carcinoma
Source: Head Neck Oncol. 2009 Jul 14;1:27. doi: 10.1186/1758-3284-1-27 (PMC2719634; doi:10.1186/1758-3284-1-27)
Supplement: Additional file 1 — Gene expression changes in HNSCC compared to normal tonsils. Data show up-regulated genes, with p-value < 0.01 and log2 ratio ≥ 1, in HNSCC compared with normal tonsil tissues. [file 1758-3284-1-27-S1.doc]

**Additional file 1**

**Title: Gene expression changes in HNSCC compared to normal tonsils.**

**Description:** Data show up-regulated genes, with *p*-value < 0.01 and log2 ratio > 1, in HNSCC compared with normal tonsil tissues.

| **Gene** | **UniGene** | ***p*-Value** | **Log2 Ratio** | **Description** | |
| --- | --- | --- | --- | --- | --- |
| **HSPB1** | Hs.76067 | **0.0038852** | **4.24375** | heat shock 27kDa protein 1 (HSPB1), mRNA. | |
| **RPLP0** | Hs.443796 | **0.0106924** | **4.05908** | ribosomal protein, large, P0 (RPLP0), transcript variant 1, mRNA. | |
| **KRT19** | Hs.309517 | **0.0002617** | **3.93486** | keratin 19 (KRT19), mRNA. | |
| **RPL37A** | Hs.433701 | **0.0077779** | **3.90159** | ribosomal protein L37a (RPL37A), mRNA. | |
| **FABP5** | Hs.408061 | **7.09E-05** | **3.75947** | fatty acid binding protein 5 (psoriasis-associated) (FABP5), mRNA. | |
| **S100A2** | Hs.515713 | **0.0001443** | **3.60879** | S100 calcium binding protein A2 (S100A2), mRNA. | |
| **ENO1** | Hs.433455 | **7.72E-05** | **3.25714** | enolase 1, (alpha) (ENO1), mRNA. | |
| **UBC** | Hs.183704 | **0.0003093** | **3.23447** | ubiquitin C (UBC), mRNA. | |
| **EIF4G2** | Hs.183684 | **0.0046831** | **3.21349** | eukaryotic translation initiation factor 4 gamma, 2 (EIF4G2), mRNA. | |
| **LAMR1** | Hs.374553 | **0.0057214** | **3.20425** | laminin receptor 1 (ribosomal protein SA, 67kDa) (LAMR1), mRNA. | |
| **RPL10** | Hs.401929 | **0.0004306** | **3.14864** | ribosomal protein L10 (RPL10), mRNA. | |
| **MIF** | Hs.407995 | **1.64E-05** | **3.08642** | macrophage migration inhibitory factor (glycosylation-inhibiting factor) (MIF), mRNA. | |
| **RPS18** | Hs.275865 | **0.0045772** | **3.065** | ribosomal protein S18 (RPS18), mRNA. | |
| **RPS3A** | Hs.356572 | **4.67E-06** | **3.05735** | ribosomal protein S3A (RPS3A), mRNA. | |
| **RPL35A** | Hs.289093 | **2.67E-05** | **2.99455** | ribosomal protein L35a (RPL35A), mRNA. | |
| **RPL21** | Hs.381123 | **0.0017345** | **2.88314** | ribosomal protein L21 (RPL21), mRNA. | |
| **PPIA** | Hs.356331 | **4.38E-05** | **2.86874** | peptidylprolyl isomerase A (cyclophilin A) (PPIA), transcript variant 1, mRNA. | |
| **TMSB10** | Hs.446574 | **0.0001183** | **2.84674** | thymosin, beta 10 (TMSB10), mRNA. | |
| **RPL18** | Hs.409634 | **3.08E-06** | **2.75531** | ribosomal protein L18 (RPL18), mRNA. | |
| **CD74** | Hs.446471 | **0.0089116** | **2.645** | CD74 antigen (invariant polypeptide of major histocompatibility complex, class II antigen-associated) | |
| **PKM2** | Hs.198281 | **9.27E-05** | **2.63368** | pyruvate kinase, muscle (PKM2), transcript variant 1, mRNA. | |
| **ATP1B3** | Hs.76941 | **0.0001585** | **2.60876** | ATPase, Na+/K+ transporting, beta 3 polypeptide (ATP1B3), mRNA. | |
| **DNCL1** | Hs.5120 | **8.39E-05** | **2.52088** | dynein, cytoplasmic, light polypeptide 1 (DNCL1), mRNA. | |
| **RPL10A** | Hs.448396 | **0.000633** | **2.50399** | ribosomal protein L10a (RPL10A), mRNA. | |
| **FTL** | Hs.433670 | **0.001325** | **2.48667** | ferritin, light polypeptide (FTL), mRNA. | |
| **KRT14** | Hs.355214 | **0.0014589** | **2.48161** | keratin 14 (epidermolysis bullosa simplex, Dowling-Meara, Koebner) (KRT14), mRNA. | |
| **RPL36AL** | Hs.444749 | **0.0002212** | **2.46479** | ribosomal protein L36a-like (RPL36AL), mRNA. | |
| **NDRG1** | Hs.318567 | **0.0003026** | **2.41031** | N-myc downstream regulated gene 1 (NDRG1), mRNA. | |
| **S100A11** | Hs.417004 | **0.0002599** | **2.37629** | S100 calcium binding protein A11 (calgizzarin) (S100A11), mRNA. | |
| **CBX3** | Hs.381189 | **0.0023134** | **2.35743** | chromobox homolog 3 (HP1 gamma homolog, Drosophila) (CBX3), transcript variant 1, mRNA. | |
| **KRT15** | Hs.80342 | **0.0036429** | **2.3412** | keratin 15 (KRT15), mRNA. | |
| **K-ALPHA-1** | Hs.446608 | **0.0004326** | **2.33306** | tubulin, alpha, ubiquitous (K-ALPHA-1), mRNA. | |
| **EEF1D** | Hs.334798 | **1.46E-06** | **2.3268** | eukaryotic translation elongation factor 1 delta (guanine nucleotide exchange protein) (EEF1D), transcript variant 2, mRNA. | |
| **CFL1** | Hs.170622 | **0.0023795** | **2.30128** | cofilin 1 (non-muscle) (CFL1), mRNA. | |
| **ARPC2** | Hs.83583 | **3.72E-05** | **2.27397** | actin related protein 2/3 complex, subunit 2, 34kDa | |
| **NME2** | Hs.433416 | **2.57E-05** | **2.26257** | non-metastatic cells 2, protein (NM23B) expressed in (NME2), mRNA. | |
| **GRN** | Hs.180577 | **2.58E-05** | **2.26029** | granulin (GRN), mRNA. | |
| **SFN** | Hs.184510 | **0.0002561** | **2.25413** | stratifin (SFN), mRNA. | |
| **EIF4A2** | Hs.511904 | **0.000112** | **2.2373** | eukaryotic translation initiation factor 4A, isoform 2 (EIF4A2), mRNA. | |
| **SQSTM1** | Hs.182248 | **2.91E-05** | **2.17552** | sequestosome 1 (SQSTM1), mRNA. | |
| **RPS26** | Hs.480569 | **0.0058472** | **2.15455** | ribosomal protein S26 (RPS26), mRNA. | |
| **PRDX1** | Hs.180909 | **0.0002097** | **2.1241** | peroxiredoxin 1 (PRDX1), transcript variant 3, mRNA. | |
| **RPS16** | Hs.397609 | **0.0001789** | **2.10157** | ribosomal protein S16 (RPS16), mRNA. | |
| **ATP5H** | Hs.155728 | **0.0015872** | **2.09749** | ATP synthase, H+ transporting, mitochondrial F0 complex, subunit d | |
| **DHCR24** | Hs.75616 | **0.0003896** | **2.087** | 24-dehydrocholesterol reductase (DHCR24), mRNA. | |
| **CCT8** | Hs.416211 | **0.009921** | **2.06184** | chaperonin containing TCP1, subunit 8 (theta) (CCT8), mRNA. | |
| **EIF3S8** | Hs.388163 | **0.000288** | **2.06097** | eukaryotic translation initiation factor 3, subunit 8, 110kDa (EIF3S8), mRNA. | |
| **CCT4** | Hs.374334 | **0.0001562** | **2.04949** | chaperonin containing TCP1, subunit 4 (delta) (CCT4), mRNA. | |
| **TEGT** | Hs.35052 | **0.0011336** | **2.02958** | testis enhanced gene transcript (BAX inhibitor 1) (TEGT), mRNA. | |
| **CTSB** | Hs.135226 | **0.0010309** | **2.02432** | cathepsin B (CTSB), transcript variant 1, mRNA. | |
| **HMGN2** | Hs.181163 | **0.000267** | **2.0068** | high-mobility group nucleosomal binding domain 2 (HMGN2), mRNA. | |
| **GPX1** | Hs.76686 | **0.0001357** | **1.98738** | glutathione peroxidase 1 (GPX1), transcript variant 1, mRNA. | |
| **PRNP** | Hs.438582 | **9.86E-05** | **1.9428** | prion protein (p27-30) (Creutzfeld-Jakob disease, Gerstmann-Strausler-Scheinker syndrome, fatal familial insomnia) (PRNP), transcript variant 2, mRNA. | |
| **CTSD** | Hs.121575 | **0.0026765** | **1.94096** | cathepsin D (lysosomal aspartyl protease) (CTSD), mRNA. | |
| **S100A14** | Hs.288998 | **0.0006713** | **1.93898** | S100 calcium binding protein A14 (S100A14), mRNA. | |
| **COX6A1** | Hs.180714 | **0.000822** | **1.93121** | cytochrome c oxidase subunit VIa polypeptide 1 (COX6A1), nuclear gene encoding mitochondrial protein, mRNA. | |
| **CANX** | Hs.155560 | **0.0018322** | **1.91926** | calnexin |  |
| **RHOA** | Hs.77273 | **0.0001489** | **1.91864** | ras homolog gene family, member A (RHOA), mRNA. | |
| **HINT1** | Hs.256697 | **5.01E-05** | **1.90289** | histidine triad nucleotide binding protein 1 (HINT1), mRNA. | |
| **SHFM1** | Hs.333495 | **0.0038872** | **1.89138** | split hand/foot malformation (ectrodactyly) type 1 (SHFM1), mRNA. | |
| **JUP** | Hs.2340 | **0.0020896** | **1.88447** | junction plakoglobin (JUP), transcript variant 2, mRNA. | |
| **IGKC** | Hs.512131 | **0.0005311** | **1.88441** | immunoglobulin kappa constant | |
| **APOE** | Hs.110675 | **0.0003442** | **1.86187** | apolipoprotein E (APOE), mRNA. | |
| **ARHGDIA** | Hs.159161 | **0.0074583** | **1.85383** | Rho GDP dissociation inhibitor (GDI) alpha | |
| **PSAP** | Hs.406455 | **0.0004024** | **1.84522** | prosaposin (variant Gaucher disease and variant metachromatic leukodystrophy) (PSAP), mRNA. | |
| **COX6B** | Hs.431668 | **6.62E-05** | **1.84165** | cytochrome c oxidase subunit VIb (COX6B), mRNA. | |
| **HMGA1** | Hs.57301 | **0.0004635** | **1.83144** | high mobility group AT-hook 1 (HMGA1), transcript variant 5, mRNA. | |
| **CST3** | Hs.304682 | **9.73E-06** | **1.82386** | cystatin C (amyloid angiopathy and cerebral hemorrhage) (CST3), mRNA. | |
| **PI3** | Hs.112341 | **0.0026898** | **1.80667** | protease inhibitor 3, skin-derived (SKALP) (PI3), mRNA. | |
| **SEC61G** | Hs.9950 | **0.0002663** | **1.79757** | Sec61 gamma subunit (SEC61G), mRNA. | |
| **COX4I1** | Hs.433419 | **0.0011239** | **1.78497** | cytochrome c oxidase subunit IV isoform 1 (COX4I1), mRNA. | |
| **ATP5B** | Hs.406510 | **0.0060241** | **1.77831** | ATP synthase, H+ transporting, mitochondrial F1 complex, beta polypeptide | |
| **HSGP25L2G** | Hs.279929 | **1.23E-05** | **1.7775** | gp25L2 protein (HSGP25L2G), mRNA. | |
| **KRT18** | Hs.406013 | **0.0097674** | **1.76784** | keratin 18 (KRT18), transcript variant 2, mRNA. | |
| **DDX17** | Hs.250696 | **0.0033104** | **1.76563** | DEAD (Asp-Glu-Ala-Asp) box polypeptide 17 (DDX17), transcript variant 2, mRNA. | |
| **AHSG** | Hs.324746 | **0.0046949** | **1.75583** | alpha-2-HS-glycoprotein (AHSG), mRNA. | |
| **LDHB** | Hs.234489 | **0.0007349** | **1.73472** | lactate dehydrogenase B (LDHB), mRNA. | |
| **ZNF505** | Hs.515284 | **0.0014791** | **1.71111** | zinc finger protein 505 (ZNF505), mRNA. | |
| **VAPA** | Hs.165195 | **0.0001842** | **1.6956** | VAMP (vesicle-associated membrane protein)-associated protein A, 33kDa (VAPA), transcript variant 2, mRNA. | |
| **PIP5K1A** | Hs.149255 | **0.0006274** | **1.68226** | phosphatidylinositol-4-phosphate 5-kinase, type I, alpha (PIP5K1A), mRNA. | |
| **KRT6C** | Hs.367762 | **0.0008503** | **1.68029** | keratin 6C (KRT6C), mRNA. | |
| **PPP2CA** | Hs.91773 | **0.0096034** | **1.66676** | protein phosphatase 2 (formerly 2A), catalytic subunit, alpha isoform (PPP2CA), mRNA. | |
| **GPNMB** | Hs.389964 | **0.0047244** | **1.65218** | glycoprotein (transmembrane) nmb (GPNMB), mRNA. | |
| **CDKN1A** | Hs.370771 | **8.92E-05** | **1.65144** | cyclin-dependent kinase inhibitor 1A (p21, Cip1) (CDKN1A), transcript variant 1, mRNA. | |
| **PSMB5** | Hs.422990 | **0.0002851** | **1.6435** | proteasome (prosome, macropain) subunit, beta type, 5 (PSMB5), mRNA. | |
| **GLUL** | Hs.442669 | **0.0004411** | **1.63248** | glutamate-ammonia ligase (glutamine synthase) (GLUL), mRNA. | |
| **TBCA** | Hs.355627 | **0.001883** | **1.62209** | tubulin-specific chaperone a (TBCA), mRNA. | |
| **GRIM19** | Hs.279574 | **0.0001906** | **1.62055** | cell death-regulatory protein GRIM19 (GRIM19), mRNA. | |
| **ADM** | Hs.441047 | **7.60E-05** | **1.61551** | adrenomedullin (ADM), mRNA. | |
| **EEF1B2** | Hs.421608 | **0.0036116** | **1.61325** | eukaryotic translation elongation factor 1 beta 2 (EEF1B2), transcript variant 2, mRNA. | |
| **PPM1G** | Hs.17883 | **0.0012217** | **1.6107** | protein phosphatase 1G (formerly 2C), magnesium-dependent, gamma isoform (PPM1G), transcript variant 2, mRNA. | |
| **G22P1** | Hs.169744 | **0.0001601** | **1.60648** | thyroid autoantigen 70kDa (Ku antigen) (G22P1), mRNA. | |
| **NEO1** | Hs.388613 | **0.0075368** | **1.59779** | neogenin homolog 1 (chicken) (NEO1), mRNA. | |
| **HNRPAB** | Hs.81361 | **0.0004683** | **1.56686** | heterogeneous nuclear ribonucleoprotein A/B (HNRPAB), transcript variant 2, mRNA. | |
| **COX5B** | Hs.1342 | **0.0002125** | **1.56069** | cytochrome c oxidase subunit Vb (COX5B), mRNA. | |
| **ATP5G3** | Hs.429 | **0.0013314** | **1.56017** | ATP synthase, H+ transporting, mitochondrial F0 complex, subunit c (subunit 9) isoform 3 (ATP5G3), nuclear gene encoding mitochondrial protein, transcript variant 3, mRNA. | |
| **LRRFIP1** | Hs.512387 | **0.0003849** | **1.53672** | leucine rich repeat (in FLII) interacting protein 1 (LRRFIP1), mRNA. | |
| **PIM2** | Hs.80205 | **8.52E-05** | **1.52674** | pim-2 oncogene (PIM2), mRNA. | |
| **HNRPL** | Hs.446623 | **0.0006186** | **1.51585** | heterogeneous nuclear ribonucleoprotein L (HNRPL), mRNA. | |
| **IFI30** | Hs.14623 | **0.0048335** | **1.50037** | interferon, gamma-inducible protein 30 | |
| **DNAJA1** | Hs.388392 | **0.0060959** | **1.50008** | DnaJ (Hsp40) homolog, subfamily A, member 1 | |
| **MT1X** | Hs.374950 | **0.0109227** | **1.4916** | metallothionein 1X (MT1X), mRNA. | |
| **ATP6C** | Hs.389107 | **0.0005107** | **1.48276** | ATPase, H+ transporting, lysosomal (vacuolar proton pump) 16kD | |
| **HNRPA1** | Hs.356721 | **0.0016031** | **1.47504** | heterogeneous nuclear ribonucleoprotein A1 (HNRPA1), transcript variant 1, mRNA. | |
| **NDUFB5** | Hs.19236 | **0.0046322** | **1.45934** | NADH dehydrogenase (ubiquinone) 1 beta subcomplex, 5, 16kDa (NDUFB5), nuclear gene encoding mitochondrial protein, mRNA. | |
| **UBE2V1** | Hs.381025 | **0.0012012** | **1.45254** | ubiquitin-conjugating enzyme E2 variant 1 (UBE2V1), transcript variant 3, mRNA. | |
| **FNTA** | Hs.356463 | **0.0001827** | **1.4381** | farnesyltransferase, CAAX box, alpha (FNTA), mRNA. | |
| **PTBP1** | Hs.172550 | **0.0031936** | **1.43575** | polypyrimidine tract binding protein 1 (PTBP1), transcript variant 3, mRNA. | |
| **DAP13** | Hs.44163 | **0.000515** | **1.43284** | 13kDa differentiation-associated protein (DAP13), mRNA. | |
| **ATF4** | Hs.181243 | **0.0007709** | **1.42755** | activating transcription factor 4 (tax-responsive enhancer element B67) (ATF4), transcript variant 2, mRNA. | |
| **FKBP1A** | Hs.374638 | **0.0039718** | **1.41968** | FK506 binding protein 1A, 12kDa (FKBP1A), transcript variant 12B, mRNA. | |
| **MRPL12** | Hs.109059 | **0.0006505** | **1.41842** | mitochondrial ribosomal protein L12 (MRPL12), nuclear gene encoding mitochondrial protein, mRNA. | |
| **CD24** | Hs.375108 | **0.0042361** | **1.41709** | CD24 antigen (small cell lung carcinoma cluster 4 antigen) (CD24), mRNA. | |
| **CLIC1** | Hs.414565 | **0.0001926** | **1.41383** | chloride intracellular channel 1 (CLIC1), mRNA. | |
| **IGFBP7** | Hs.435795 | **0.0090176** | **1.40922** | insulin-like growth factor binding protein 7 (IGFBP7), mRNA. | |
| **TNFRSF1A** | Hs.159 | **0.0104552** | **1.40822** | tumor necrosis factor receptor superfamily, member 1A (TNFRSF1A), mRNA. | |
| **IL26** | Hs.272350 | **7.74E-05** | **1.40663** | interleukin 26 (IL26), mRNA. | |
| **PITX1** | Hs.84136 | **0.0071843** | **1.39906** | paired-like homeodomain transcription factor 1 (PITX1), mRNA. | |
| **IGFBP2** | Hs.433326 | **0.0050698** | **1.39064** | insulin-like growth factor binding protein 2 (36kD) (IGFBP2), mRNA. | |
| **TRIM28** | Hs.433612 | **0.0095368** | **1.38605** | tripartite motif-containing 28 (TRIM28), mRNA. | |
| **FBXO7** | Hs.5912 | **0.003777** | **1.37933** | F-box protein 7 (FBXO7), mRNA. | |
| **ARPC3** | Hs.439511 | **0.0007252** | **1.37398** | actin related protein 2/3 complex, subunit 3, 21kDa (ARPC3), mRNA. | |
| **SDC1** | Hs.82109 | **0.0008439** | **1.36921** | syndecan 1 (SDC1), mRNA. | |
| **GTF2I** | Hs.408507 | **0.0001233** | **1.36301** | general transcription factor II, i (GTF2I), transcript variant 3, mRNA. | |
| **DAZAP2** | Hs.369761 | **0.0006157** | **1.36076** | DAZ associated protein 2 (DAZAP2), mRNA. | |
| **SEMA3F** | Hs.32981 | **0.0086229** | **1.35433** | sema domain, immunoglobulin domain (Ig), short basic domain, secreted, (semaphorin) 3F (SEMA3F), mRNA. | |
| **IDH2** | Hs.5337 | **0.0013234** | **1.34547** | isocitrate dehydrogenase 2 (NADP+), mitochondrial (IDH2), mRNA. | |
| **CHD4** | Hs.74441 | **0.0002637** | **1.34457** | chromodomain helicase DNA binding protein 4 (CHD4), mRNA. | |
| **FBL** | Hs.99853 | **0.0109811** | **1.3118** | fibrillarin |  |
| **CD164** | Hs.43910 | **0.0098992** | **1.31139** | CD164 antigen, sialomucin (CD164), mRNA. | |
| **PRKDC** | Hs.415749 | **0.0058541** | **1.30404** | protein kinase, DNA-activated, catalytic polypeptide (PRKDC), mRNA. | |
| **IL1RN** | Hs.81134 | **0.0004638** | **1.30338** | interleukin 1 receptor antagonist (IL1RN), transcript variant 1, mRNA. | |
| **PKMYT1** | Hs.77783 | **0.005362** | **1.29542** | membrane-associated tyrosine- and threonine-specific cdc2-inhibitory kinase (PKMYT1), transcript variant 1, mRNA. | |
| **SEPW1** | Hs.433941 | **0.0012975** | **1.28794** | selenoprotein W, 1 (SEPW1), mRNA. | |
| **TKT** | Hs.89643 | **0.008019** | **1.28711** | transketolase (Wernicke-Korsakoff syndrome) (TKT), mRNA. | |
| **LAMP1** | Hs.150101 | **0.0078982** | **1.285** | lysosomal-associated membrane protein 1 (LAMP1), mRNA. | |
| **RPA2** | Hs.79411 | **0.0003362** | **1.28499** | replication protein A2, 32kDa (RPA2), mRNA. | |
| **PRPF8** | Hs.181368 | **0.0015648** | **1.25988** | PRP8 pre-mRNA processing factor 8 homolog (yeast) (PRPF8), mRNA. | |
| **VMP1** | Hs.166254 | **0.0052713** | **1.25343** | likely ortholog of rat vacuole membrane protein 1 (VMP1), mRNA. | |
| **FUS** | Hs.107720 | **0.0015828** | **1.25019** | fusion (involved in t(12;16) in malignant liposarcoma) (FUS), mRNA. | |
| **UNQ6077** | Hs.428779 | **1.07E-05** | **1.24796** | AAAP6077 |  |
| **SHCBP1** | Hs.123253 | **0.0008927** | **1.2465** | SHC SH2-domain binding protein 1 (SHCBP1), mRNA. | |
| **MKNK2** | Hs.512094 | **0.0073385** | **1.24559** | MAP kinase-interacting serine/threonine kinase 2 (MKNK2), mRNA. | |
| **IRF6** | Hs.655625 | **0.0007456** | **1.23742** | interferon regulatory factor 6 | |
| **DDOST** | Hs.301882 | **0.0027428** | **1.23463** | dolichyl-diphosphooligosaccharide-protein glycosyltransferase (DDOST), mRNA. | |
| **POLR2A** | Hs.171880 | **1.43E-05** | **1.21974** | polymerase (RNA) II (DNA directed) polypeptide A, 220kDa (POLR2A), mRNA. | |
| **PEA15** | Hs.194673 | **0.0004502** | **1.21833** | phosphoprotein enriched in astrocytes 15 (PEA15), mRNA. | |
| **FADD** | Hs.86131 | **0.0022123** | **1.20889** | Fas (TNFRSF6)-associated via death domain (FADD), mRNA. | |
| **HYPK** | Hs.511978 | **0.007262** | **1.2079** | Huntingtin interacting protein K (HYPK), mRNA. | |
| **DRE1** | Hs.246875 | **0.0075827** | **1.20266** | DRE1 protein |  |
| **S100A13** | Hs.446592 | **0.0002925** | **1.19819** | S100 calcium binding protein A13 (S100A13), mRNA. | |
| **ANXA11** | Hs.75510 | **0.0032852** | **1.19793** | annexin A11 (ANXA11), transcript variant a, mRNA. | |
| **GPC1** | Hs.328232 | **0.0005777** | **1.19381** | glypican 1 (GPC1), mRNA. | |
| **SIAHBP1** | Hs.74562 | **0.0057071** | **1.18636** | fuse-binding protein-interacting repressor (SIAHBP1), transcript variant 2, mRNA. | |
| **HEXB** | Hs.69293 | **0.0062792** | **1.18544** | hexosaminidase B (beta polypeptide) (HEXB), mRNA. | |
| **ADIPOR2** | Hs.334854 | **0.0029571** | **1.18417** | adiponectin receptor 2 (ADIPOR2), mRNA. | |
| **COX17** | Hs.16297 | **0.0005802** | **1.18278** | COX17 homolog, cytochrome c oxidase assembly protein (yeast) (COX17), nuclear gene encoding mitochondrial protein, mRNA. | |
| **POLR2I** | Hs.47062 | **0.0026696** | **1.1765** | polymerase (RNA) II (DNA directed) polypeptide I, 14.5kDa (POLR2I), mRNA. | |
| **STARD7** | Hs.445446 | **0.001792** | **1.17616** | START domain containing 7 (STARD7), transcript variant 2, mRNA. | |
| **SH3MD2** | Hs.301804 | **0.0002364** | **1.16288** | SH3 multiple domains 2 (SH3MD2), mRNA. | |
| **PSMD4** | Hs.505059 | **0.0036839** | **1.15227** | proteasome (prosome, macropain) 26S subunit, non-ATPase, 4 | |
| **EMP1** | Hs.306692 | **0.0017771** | **1.15085** | epithelial membrane protein 1 (EMP1), mRNA. | |
| **F11R** | Hs.506845 | **0.0041912** | **1.14801** | F11 receptor (F11R), transcript variant 2, mRNA. | |
| **SSR4** | Hs.409223 | **0.0003293** | **1.14407** | signal sequence receptor, delta (translocon-associated protein delta) (SSR4), mRNA. | |
| **PBP** | Hs.433863 | **0.0006439** | **1.14277** | prostatic binding protein (PBP), mRNA. | |
| **HIF1A** | Hs.412416 | **0.0023125** | **1.14196** | hypoxia-inducible factor 1, alpha subunit (basic helix-loop-helix transcription factor) (HIF1A), transcript variant 2, mRNA. | |
| **PPP4C** | Hs.2903 | **0.0004758** | **1.13512** | protein phosphatase 4 (formerly X), catalytic subunit (PPP4C), mRNA. | |
| **SAP30L** | Hs.120425 | **1.24E-05** | **1.13408** | Sin3A associated protein p30-like (SAP30L), mRNA. | |
| **SLC3A2** | Hs.79748 | **0.0073272** | **1.132** | solute carrier family 3 (activators of dibasic and neutral amino acid transport), member 2 (SLC3A2), mRNA. | |
| **SERPINB6** | Hs.41072 | **0.0001029** | **1.13142** | serine (or cysteine) proteinase inhibitor, clade B (ovalbumin), member 6 (SERPINB6), mRNA. | |
| **SET** | Hs.436687 | **0.0082682** | **1.13026** | SET translocation (myeloid leukemia-associated) (SET), mRNA. | |
| **CBR1** | Hs.88778 | **0.0031672** | **1.12608** | carbonyl reductase 1 (CBR1), mRNA. | |
| **PMS2L9** | Hs.278467 | **0.0099769** | **1.12595** | postmeiotic segregation increased 2-like 9 (PMS2L9), mRNA. | |
| **PTPLB** | Hs.5957 | **0.0038647** | **1.12054** | protein tyrosine phosphatase-like (proline instead of catalytic arginine), member b (PTPLB), mRNA. | |
| **IMPDH2** | Hs.75432 | **0.0101007** | **1.11607** | IMP (inosine monophosphate) dehydrogenase 2 (IMPDH2), mRNA. | |
| **EIF3S4** | Hs.28081 | **0.0068331** | **1.07669** | eukaryotic translation initiation factor 3, subunit 4 delta, 44kDa (EIF3S4), mRNA. | |
| **NOLA2** | Hs.386392 | **0.0073027** | **1.06915** | nucleolar protein family A, member 2 (H/ACA small nucleolar RNPs) (NOLA2), mRNA. | |
| **ANXA8** | Hs.87268 | **0.0018229** | **1.06741** | annexin A8 (ANXA8), mRNA. | |
| **SGKL** | Hs.380877 | **0.0082469** | **1.06202** | serum/glucocorticoid regulated kinase-like (SGKL), transcript variant 2, mRNA. | |
| **HLA-DPB1** | Hs.368409 | **0.0098907** | **1.04708** | major histocompatibility complex, class II, DP beta 1 (HLA-DPB1), mRNA. | |
| **SERPINB4** | Hs.123035 | **0.0019845** | **1.04391** | serine (or cysteine) proteinase inhibitor, clade B (ovalbumin), member 4 (SERPINB4), mRNA. | |
| **NFKB2** | Hs.73090 | **0.0036939** | **1.04079** | nuclear factor of kappa light polypeptide gene enhancer in B-cells 2 (p49/p100) (NFKB2), mRNA. | |
| **CAMK2B** | Hs.321572 | **0.0018886** | **1.03602** | calcium/calmodulin-dependent protein kinase (CaM kinase) II beta | |
| **HAX1** | Hs.199625 | **0.0001441** | **1.03101** | HS1 binding protein (HAX1), mRNA. | |
| **PRKAG1** | Hs.3136 | **0.001114** | **1.02461** | protein kinase, AMP-activated, gamma 1 non-catalytic subunit (PRKAG1), transcript variant 1, mRNA. | |
| **DHFR** | Hs.83765 | **0.0058204** | **1.02459** | dihydrofolate reductase (DHFR), mRNA. | |
| **MBC2** | Hs.8309 | **0.000152** | **1.01989** | likely ortholog of mouse membrane bound C2 domain containing protein (MBC2), mRNA. | |
| **PRDX6** | Hs.120 | **0.0071425** | **1.01627** | peroxiredoxin 6 (PRDX6), mRNA. | |
| **CAPZA2** | Hs.369579 | **0.0023007** | **1.00984** | capping protein (actin filament) muscle Z-line, alpha 2 (CAPZA2), mRNA. | |
| **DUSP1** | Hs.171695 | **0.0020348** | **1.00859** | dual specificity phosphatase 1 (DUSP1), mRNA. | |
| **LRP1** | Hs.162757 | **0.0017459** | **1.00654** | low density lipoprotein-related protein 1 (alpha-2-macroglobulin receptor) (LRP1), mRNA. | |
| **HSPG2** | Hs.211573 | **0.0018552** | **1.00488** | heparan sulfate proteoglycan 2 (perlecan) | |
| **PLEKHJ1** | Hs.512626 | **0.0003142** | **1.00408** | pleckstrin homology domain containing, family J member 1 (PLEKHJ1), mRNA. | |
